# Exploring Consumer Palatability of Australian Beef Fajita Meat Enhanced with Phosphate or Sodium Bicarbonate

**DOI:** 10.3390/foods9020177

**Published:** 2020-02-11

**Authors:** Andrea Garmyn, Nicholas Hardcastle, Clay Bendele, Rod Polkinghorne, Mark Miller

**Affiliations:** 1 Department of Animal and Food Sciences, Texas Tech University, Lubbock, TX 79409, USA; nicholas.hardcastle@ttu.edu (N.H.); clay.bendele@ttu.edu (C.B.); mfmrraider@aol.com (M.M.); 2 Birkenwood Pty. Ltd., 46 Church St, Hawthorn VIC 3122, Australia; rod.polkinghorne@gmail.com

**Keywords:** beef, consumer, eating quality, enhancement, fajita, muscle, phosphate, sensory testing, sodium bicarbonate

## Abstract

The objective of this study was to determine the consumer eating quality of five Australian beef muscles (outside skirt/*diaphragm*, inside skirt/*transversus abdominis*, inside round cap/*gracilis*, bottom sirloin flap/*obliquus abdominis internus*, and flank steak/*rectus abdominis*) served as fajita strips. All the muscles were divided in half and enhanced (12%) with a brine solution containing either phosphate, a “clean label” ingredient sodium bicarbonate, or not enhanced. Muscle and enhancement independently influenced (*P* < 0.01) tenderness, juiciness, flavor, and overall liking. Overall, the bottom sirloin flap was liked the most (*P* < 0.05) when compared with all the other muscles, while the inside round cap was liked less but did not differ (*P* > 0.05) from the inside skirt or flank steak. Samples enhanced with sodium bicarbonate were the most (*P* < 0.05) tender and juicy; samples enhanced with phosphate were intermediate, and the control samples were the least tender and juicy, regardless of the muscle. Flavor and overall liking were similar (*P* > 0.05) between clean and phosphate-enhanced samples, and both were liked more than the control samples. Enhancement was necessary for acceptable eating quality of all the muscles evaluated in this study; however, the inside round cap was the least suitable. These results indicate that a “clean label” enhanced fajita product is possible without compromising cooking yield or consumer satisfaction.

## 1. Introduction

The global beef industry strives to produce and deliver consistent, high-quality beef products that meet expectations for consumer eating quality. In Australia, beef eating quality is underpinned by the Meat Standards Australia (MSA) grading system [1], but this standard focuses on predicting the eating quality of fresh beef (i.e., no enhancement). Garmyn et al. [2] recently showed that enhancement significantly improved palatability in Australian *longissimus lumborum* and *gluteus medius* steaks with greater consumer ratings for tenderness, juiciness, flavor liking, overall liking, and satisfaction according to US consumers. Likewise, Lees et al. [3] found that infusion with kiwifruit extract improved consumer scores for eating quality of the *longissimus lumborum* and *biceps femoris*.

Value-adding or enhancement is still required of some lower quality cuts or cuts that would otherwise be considered unsatisfactory in the MSA grading system. Many researchers have explored the use of non-meat ingredients, such as phosphate and salt, to enhance beef quality and sensory characteristics [2,4,5,6,7,8]. These studies examined the enhancement of beef *longissimus dorsi*, muscles from the chuck (*complexus*, *serratus ventralis*, *splenius*, *subscapularis*, *supraspinatus*, and *triceps brachii*), *biceps femoris*, and *gluteus medius*, but the investigation of enhancement in beef muscles outside this list is somewhat limited.

Beef fajitas are commonly found on menus of Mexican restaurants in the US and utilize muscles, such as the inside or outside skirt steak or the flank steak, combined with marinade or seasoning. Huerta-Montauti et al. [9] investigated processing techniques of several muscles for thin meat alternatives, including the outside and inside skirt steaks for fajitas, but limited research has been done exploring the use of enhancement on flank steaks [10] or other muscles from the round or sirloin.

Given the consumer interest in clean labeling [11], finding an alternative functional ingredient that can be used in lieu of phosphate for meat enhancement also merits investigation. Studies have investigated the use of sodium bicarbonate (NaHCO_3−_) as a phosphate replacement in pork [12] and chicken [13]. These studies have shown that NaHCO_3_ can positively affect the physiochemical and eating quality attributes of meat and poultry compared with non-treated counterparts. In an attempt to classify phosphate alternatives’ impact on beef eating quality, Hardcastle et al. [14] determined that Australian steaks from multiple muscles enhanced with NaHCO_3−_ resulted in greater consumer palatability scores compared with those treated with sodium phosphate. Value-adding lower-quality muscles are of extreme interest, particularly if muscles destined for beef trim could be utilized in dishes such as fajitas or stir fry rather than ground beef. Therefore, our objective was to determine the consumer eating quality of five Australian beef muscles selected from carcasses representative of four MSA-predicted eating quality grades. Muscles were enhanced (12%) with either phosphate, sodium bicarbonate, or not enhanced. Ultimately, we investigated which of these muscles are suitable for fajita manufacture and examined the usefulness of the MSA sorting system for these muscles when using a preparation/cook method previously untested in the MSA grading scheme [15]. We hypothesized that differences in eating quality would exist between muscles and enhancement treatments, but that enhancement would mitigate any differences in eating quality due to the MSA grade.

## 2. Materials and Methods

### 2.1. Product Procurement

Cattle were harvested on one of four days within a two-week period at a commercial abattoir in Rockhampton, Australia. Tropical breed content and implant usage was monitored and reported for cattle according to their accompanying MSA vendor declaration when they were transferred to an MSA-licensed abattoir [16]. All cattle had substantial and equal *Bos indicus* influence, none had received hormone growth promotants, approximately 36% were grass-fed and 63% were grain-fed, and 60% were male and 40% of cattle were female. Carcasses were subjected to MSA grading, and data were recorded and utilized for sorting purposes. MSA marbling was scored from 100 to 1190 in increments of 10 based on the amount and distribution of marbling in the longissimus dorsi [17]. Ossification was scored from 100 to 590 in increments of 10 using the AUS-MEAT Carcase Maturity Chart [17]. In addition, hot carcass weight (HCW; kg), 12th rib fat thickness (mm), eye muscle area (cm^2^), and hump height (mm) were collected and recorded for each carcass. In the MSA grading system, hump height serves as an indicator of tropical breed content. AUS-MEAT fat and meat color scores were also recorded [17]. Finally, *longissimus* muscle ultimate pH and temperature values were recorded using a hand-held, probe-type pH meter (model WP-80, TPS Pty Ltd, Springwood, Brisbane, Australia). Carcass data were used to select carcasses representative of four different predicted eating quality categories: 2 = unsatisfactory; 3* = “Selected”; 4* = “Classic”; and 5* = “Premium”.

Five subprimals were collected from the right side of each carcass: outside skirt [Institutional Meat Purchase Specification (IMPS) 121C; *diaphragm*], inside skirt (IMPS 121D; *transversus abdominis*), inside round cap (IMPS 169B; *gracilis*), bottom sirloin flap (IMPS 185A; *obliquus abdominis internus*), and flank steak (IMPS 193; *rectus abdominis*). Subprimals were vacuum packaged individually, held in chilled storage at 0 to 1 °C until 14 d postmortem, and frozen at −20 °C. All subprimals were combined into a single consignment from Brisbane, Australia, to Texas Tech University, Lubbock, TX, USA, via cargo freight and road transport. Subprimals were held at frozen temperatures (−20 °C) during shipment and storage upon arrival at Texas Tech University, Lubbock, TX, USA.

### 2.2. Sample Processing

The muscles were thawed for 48 h at 2 to 4 °C before sample processing. Excess fat and sinew were removed from all the muscles prior to processing. All the muscles were cut in half parallel to the muscle fibers, with each half being alternatively assigned to enhancement treatment (control, clean, and phosphate). All the muscle halves were weighed to obtain the green weight. The control muscle halves were untreated. The clean muscle halves were enhanced with sodium chloride (NaCl; Morton Salt Inc., Chicago, IL, USA), food grade sodium bicarbonate (NaHCO_3_; Church & Dwight Co. Inc., Ewing Township, NJ, USA), and water. The brine was prepared in 5 °C tap water with 4.16% NaCl and 4.00% NaHCO_3−_. Brine was poured over the muscles in the vacuum tumbler for a target pickup of 112% (111.68% ± 3.82) of fresh muscle weight. All the muscles were placed in a vacuum tumbler (Koch Industries, Wichita, KS, USA) and a vacuum (508 mm Hg) was pulled. The muscles were batch tumbled at 10 RPM for 20 min. The samples were allowed to rest for 10 min, and the tumbled weight was obtained and recorded for each muscle half. The pickup percentage was calculated by taking the tumbled weight divided by the green weight, multiplied by 100.

The phosphate muscle halves were enhanced with NaCl (Morton Salt Inc., Chicago, IL, USA), sodium tripolyphosphate (STPP; Carfosel 408, Prayon Inc., Augusta, GA, USA), and water. The brine was prepared in 5 °C tap water with 4.16% NaCl and 4.00% STPP. Brine was poured over the muscles in the vacuum tumbler for a target pickup of 112% (111.59% ± 4.00) of fresh muscle weight. The muscles were tumbled as previously described for clean enhancement. The samples were allowed to rest for 10 min, and the tumbled weight was obtained and recorded for each muscle half. The pickup percentage was calculated by taking the tumbled weight divided by the green weight, multiplied by 100.

Regardless of enhancement treatment, a sample was obtained (weighing approximately 10 g) after treatment application and before vacuum packaging. This sample was vacuum packaged individually and frozen at −20 °C until analysis of final pH. All the remaining muscle samples were vacuum packaged individually and held at 2 to 4 °C. The samples were sorted into one of six testing days, and the muscles were used in consumer sensory testing within 9 days of processing.

### 2.3. Ultimate pH Determination

The samples were thawed for 24 h at 2 to 4 °C prior to analysis. Individual samples were mixed with distilled water for 1 min in a tabletop blender (Model 80335R, Hamilton Beach Brands, Glen Allen, VA, USA) to allow for homogenization. Homogenized samples were placed in a 150 mL beaker with a filter cone. The sample pH was measured with a bench-top probe-type pH meter (Model 14703; Denver Instrument Company, Bohemia, NY, USA), and the ultimate pH of each sample was determined as the average of two samples.

### 2.4. Consumer Sensory Evaluation

The Texas Tech University Institutional Review Board approved procedures for use of human subjects for consumer panel evaluation of meat sensory attributes (IRB#: 2017-598).

Each muscle sample was removed from packaging and weighed individually to obtain a raw weight. The muscles were cooked individually to 74 °C on a George Foreman clamshell grill (Model GRP99, Spectrum Brands. Inc., Middleton, WI, USA) with the lid closed and a plate temperature set to 218 °C. The muscle temperature was monitored using a digital, instant read Thermapen thermometer (Model Mk4, ThermoWorks, American Fork, UT, USA). When the muscles reached the required temperature, they were removed from the heat source. The peak temperature and cooked weight were recorded. The cooking loss percentage was calculated by subtracting the cooked weight from the raw weight, dividing by the raw weight, and multiplying by 100. In addition, total cooking time was recorded. The muscles were rested for at least 3 min prior to slicing. The muscles were sliced into 13 mm strips perpendicular to the muscle fibers, and the strips were cut in half lengthwise, resulting in strips that were approximately 5 cm long. The strips were transferred to pre-heated rectangular stainless-steel pans, which were maintained in insulated water bath warming units (Model W-3Vi; American Permanent Ware Company; Dallas, TX, USA) at ~60 °C throughout the test session. Each warming unit held nine pans.

Consumer panels were conducted in the Texas Tech University Animal and Food Sciences Building. Consumer panelists (*n* = 360) were recruited from Lubbock, Texas, and the surrounding local communities by scheduling community groups with populations of regular red meat eaters within the age range of 18–75. Each consumer was monetarily compensated and was only allowed to participate one time. Each session consisted of 60 people and lasted approximately 60 min.

Consumer testing was conducted according to MSA protocols [15], with previously described modifications for cooking method. Each consumer evaluated seven samples, including one warm-up sample, which was excluded from analysis, to orient consumers to the sample format. The warm-up samples were always served in the first position, followed by six test samples served in a predetermined, balanced order. The serving order of the six test products was controlled by a 6 × 6 Latin square design, ensuring that all the products were presented an equal number of times in each serving order position and before and after each other product. The test products were selected from the five muscles that were or were not enhanced. The predicted eating quality score (MSA grade) was also used for selection and allocation into the consumer testing design. Those products were equally represented and evenly distributed among the 60 consumers each evening. The testing occurred over the course of six evenings, with a similar and even product distribution in each testing session. Software-controlled routines ensured that the samples from each individual muscle were served in five different order positions and within different subsets of 12 consumers within each group of 60.

Each panelist was seated at a numbered booth and was provided with a ballot, plastic utensils, a toothpick, unsalted crackers, a napkin, an empty cup, a water cup, and a cup with diluted apple juice (10% apple juice and 90% water). Each ballot consisted of a demographic questionnaire, seven sample ballots, and a post-panel survey regarding beef purchasing habits. Before beginning each panel, consumers were given verbal instructions by Texas Tech personnel about the ballot and the process of testing samples. The panels were conducted in a large classroom that has standard fluorescent lighting (i.e., no red filters were used) with tables that were divided into individual sensory booths.

Each sample had 10 consumer observations (i.e., each muscle half yielding at least 20 strips served in duplicate to 10 predetermined consumers). Consumers scored palatability traits, including tenderness, juiciness, flavor liking, and overall liking, on 100-mm line scales verbally anchored at 0 (not tender, not juicy, dislike extremely) and 100 (very tender, very juicy, like extremely). Consumers were asked to rate the quality of each sample as unsatisfactory, good everyday quality, better than everyday quality, or premium quality. The 10 individual scores for each trait were averaged to generate mean sensory scores for each palatability trait and satisfaction prior to analysis. A composite score (MQ4) was calculated using the following equation: (tenderness × 0.3) + (juiciness × 0.1) + (flavor liking × 0.3) + (overall liking × 0.3) [15]. Weightings for tenderness have decreased and flavor liking increased from original weightings by [15] for a balanced contribution to the MQ4 value. The weightings give an indication of the relative importance of the four sensory attributes (tenderness, juiciness, flavor, and overall satisfaction) to the final meat quality score. According to the fixed weightings above, when a consumer eats beef, their satisfaction is influenced equally by tenderness, flavor liking, and overall liking, with a lesser contribution by juiciness.

All remaining pieces that were not consumed were vacuum packaged and chilled overnight at 2–4 °C. The samples were then snap frozen in liquid nitrogen. Frozen and cubed samples were homogenized in a food processor (Model Blixer 3 Series D, Robot Coupe, Ridgeland, MS, USA), blended into an ultrafine powder, and transferred into a labeled Whirl-Pak bag. The bags were stored in a freezer at −80 °C until subsequent analysis for percent moisture.

### 2.5. Percent Moisture

Moisture percentages were obtained for the cooked samples. The cooked sample moisture percentages were obtained in accordance with AOAC International protocol #950.46 [18]. Five grams (±0.05 g) of powdered sample were weighed into crucibles. The weight of each crucible and the crucible plus the sample weight were recorded. The samples were placed in a drying oven (Isotemp Oven, Thermo Fischer Scientific, Waltham, MA, USA) at 100 °C for 24 h. Upon completion of drying, the crucibles were removed from the oven and placed into desiccators for 30 min to cool. The crucibles were then weighed to determine the percentage of moisture in each sample. The percent moisture was obtained by taking the difference between the pre- and post-dried crucibles divided by the pre-dried crucibles and multiplied by 100. The total moisture was calculated by taking the sample weight and multiplying by the percent moisture for water holding capacity determination.

### 2.6. Statistical Analysis

The data were analyzed in SAS using PROC GLIMMIX (version 9.4, SAS Inst. Inc., Cary, NC, USA). For compositional analyses, the enhancement treatment, the muscle, and their interaction were included as fixed effects. For consumer sensory analyses, the enhancement treatment, the muscle, the MSA grade, and their interactions were included as fixed effects. The percent pickup was considered and tested as a potential covariate but was decided against due to its relationship both to the muscle (one of the treatment factors) and the enhancement treatment, as that would violate the statistical guidelines for covariate usage. Treatment least squares means were separated with the PDIFF option of SAS using a significance level of *P* ≤ 0.05. Mean separation tests for all pairwise comparisons were performed using the PDIFF function, which requests that *P*-values for differences of all least squares means be produced. The PROC CORR of SAS was used to assess the relationship between compositional traits and consumer eating quality traits by generating Pearson correlation coefficients. The PROC FREQ of SAS was used to summarize consumer demographic information.

## 3. Results

### 3.1. Carcass Traits

All the carcasses were graded using MSA grading specifications. The carcass characterization can be found in Table 1.

### 3.2. Processing Characteristics

As seen in Table 2, muscle and enhancement influenced (*P* < 0.01) post-processing pH, and muscle impacted (*P* < 0.01) all other processing characteristics, but the percent pickup was not different between the clean and phosphate enhancement treatments (*P* = 0.52). No interactions between muscle and enhancement were detected (*P* ≥ 0.10) for processing characteristics.

Tumbling muscles with an enhancement solution containing sodium bicarbonate resulted in the greatest post-processing pH, phosphate-enhanced muscles had an intermediate pH, and the control samples that were not enhanced had the lowest post-processing pH (*P* < 0.05). The green weight, tumbled weight, and percent pickup were not influenced (*P* ≥ 0.52) by enhancement. Post-processing pH varied by muscle (*P* < 0.01). The bottom sirloin flap and flank steak had greater (*P* < 0.05) pH than the outside round cap or outside skirt, but flank steak and inside skirt did not differ (*P* > 0.05). For the green weight and the tumbled weight, the bottom sirloin flap samples were the heaviest (*P* < 0.05), followed by the inside round cap and inside skirt, which were intermediate; the flank steak and outside skirt had the lightest muscle weights. Although the percent pickup was similar between the enhancement treatments, the percent pickup was influenced by the muscle (*P* < 0.01). Because all the muscles were batch tumbled as opposed to tumbling by muscle, variation in the raw material size existed, which led to uneven solution uptake by the different muscles [19]. The outside skirt had the greatest (*P* < 0.05) percent pickup, followed by the bottom sirloin flap and inside skirt, which were similar (*P* > 0.05). The flank steak had the lowest percent pickup (*P* < 0.05) compared with all the other muscles.

### 3.3. Cooking Properties

Table 3 and Table 4 illustrate the effects of muscle and enhancement on the cooking properties of the beef muscle samples. Cooking loss and cooking time were both influenced (*P* ≤ 0.02) by muscle. The inside skirt had lower (*P* < 0.05) cooking loss than did the outside skirt and inside round cap but did not differ (*P* > 0.05) from the other muscles. As expected, the larger and thicker muscles took longer (*P* < 0.05) to cook than the smaller or thinner muscles. Cooking loss was impacted (*P* < 0.01) but cooking time was not influenced (*P* = 0.71) by the enhancement treatment. The muscles enhanced with phosphate had greater (*P* < 0.05) cooking loss than the clean enhancement or the control, which was somewhat unexpected given the elevated post-processing pH of the phosphate enhanced muscles compared with the pH of the control samples.

Table 4 shows the interactive effects of muscle and enhancement on the cooked moisture percentage (*P* < 0.01). For the outside skirt, inside skirt, and flank steak, the muscles tumbled with sodium bicarbonate had a greater (*P* < 0.05) cooked moisture percentage than the muscles tumbled with phosphate, which in turn had a greater (*P* < 0.05) moisture percentage than the non-enhanced control samples. For the bottom sirloin flap and inside round cap, the cooked moisture percentage did not differ (*P* > 0.05) between the samples enhanced with sodium bicarbonate or phosphate, but all the enhanced samples had a greater (*P* < 0.05) cooked moisture percentage compared with the non-enhanced control samples.

### 3.4. Consumer Sensory

The demographic characteristics of participating consumers can be found in Table 5. Over half of the participants were aged 20–39 years old. Nearly half of the population in Lubbock, TX, USA, is less than 45 years old [20], so this percentage of consumers is representative of society. We also believe this percentage is suitable according to the product studied. Participants were evenly split between male and female. Most participants (97.2%) identified with Caucasian/white or Hispanic as their ethnic origin, with an even split between the two distinctions. For census purposes, persons who identify as Hispanic or Latino can identify as any race; however, in the latest census data available for Lubbock, TX, USA, 35% reported themselves as Hispanic or Latino, while 65% reported themselves as not Hispanic or Latino [20]. The most common household size consisted of 2–3 adults, representing over 80% of participants. Nearly half of the participants had no children living in their household. The level of education with the highest proportion of participants was for “some college/technical school” (32.4%), while high school and college graduates collectively accounted for another 50%. Additionally, the majority of consumers ate beef at least twice per week (82.8%). The most preferred degree of doneness was medium-rare, with medium and medium-well contributing another 49% collectively.

The consumer sensory outcomes can be found in Table 6. No interactions were detected (*P* > 0.05) between muscle, enhancement, or MSA grade. Muscle and enhancement independently influenced (*P* < 0.01) tenderness, juiciness, flavor liking, overall liking, MQ4, and satisfaction. The MSA grade only impacted tenderness but had no effect on juiciness, flavor liking, overall liking, MQ4, or satisfaction. For tenderness and juiciness, the bottom sirloin flap was scored higher (*P* < 0.05) than any other muscle, followed by the outside skirt. The inside skirt and flank steak were scored similarly (*P* > 0.05) for tenderness and juiciness, and the inside round cap was the least (*P* < 0.05) tender and juicy compared with all the other muscles. Consumers liked the flavor of the bottom sirloin flap more (*P* < 0.05) than all other muscles, which did not differ (*P* > 0.05). Ultimately, the bottom sirloin flap was liked most (*P* < 0.05) overall compared with all other muscles, while the inside round cap was liked less, but did not differ (*P* > 0.05) from the inside skirt or flank steak. The bottom sirloin flap had the greatest composite MQ4 score, followed by the outside skirt, flank steak, and inside skirt, which were similar (*P* > 0.05) and had intermediate MQ4. Lastly, the inside round cap had the lowest (*P* < 0.05) composite MQ4 score compared with all other muscles. Satisfaction followed a similar trend to overall liking in terms of the differences between the muscles, but all muscles were classified as “good everyday quality” according to their satisfaction score.

The samples enhanced with sodium bicarbonate were the most (*P* < 0.05) tender and juicy, the samples enhanced with phosphate were intermediate, and the control samples were the least tender and juicy, regardless of muscle or MSA grade. Despite differences in tenderness and juiciness between enhanced samples, flavor and overall liking were similar (*P* > 0.05) between the clean and phosphate-enhanced samples, and both were liked more than the control samples. MQ4 score and satisfaction of all the samples increased (*P* < 0.05) due to enhancement, resulting in a shift into the next quality category for satisfaction. MQ4 and satisfaction did not differ between the clean or phosphate enhancement treatments. So, rather than being considered “unsatisfactory”, the enhanced samples were perceived as “good everyday quality”.

Lastly, the MSA grade influenced (*P* = 0.04) tenderness scores only but not in a linear fashion. The classic (4*) samples were more tender than the premium (5*) and unsatisfactory (2) samples but did not differ (*P* > 0.05) from the selected (3*) samples. These results suggest that predicted eating quality scores may need to be adjusted when value-adding strategies, such as enhancement and alternative cooking methods, are employed.

### 3.5. Correlations

Table 7 illustrates the relationships between composition and cooking characteristics with eating quality traits. All the eating traits were positively related (*P* < 0.01) with post-processing pH, suggesting that greater pH resulted in higher eating quality. A similar trend was observed for the percent pickup (*P* < 0.01). The cooked moisture was also positively correlated (*P* < 0.01) to eating quality traits; this was likely due to the greater water holding capacity induced from greater pH from enhancement. Cooking time was positively linked (*P* < 0.01) to flavor liking, suggesting that the samples that took longer to cook had longer for flavor to develop, which was valued by consumers. Lastly, cooking loss was negatively associated (*P* < 0.05) with tenderness and juiciness.

To estimate the extent to which eating quality scores are linked to overall liking and satisfaction, correlation coefficients between palatability traits, MQ4, and satisfaction scores were determined (Table 8). Consumer overall liking was associated (*P* < 0.01) with consumer tenderness (*r* = 0.88) and juiciness ratings (*r* = 0. 87) but most highly related with flavor liking (*r* = 0.96). Individual palatability traits were strongly correlated to each other (*r* ≥ 0.80), indicating that individual improvements of these traits could influence the perception of another trait. MQ4 was highly related (*P* < 0.01) to eating quality scores for tenderness, juiciness, flavor liking, and overall liking, as would be expected given that it is a composite score of those traits. Satisfaction was positively linked (*P* < 0.01) to all the eating quality traits, especially overall liking, and was highly correlated to MQ4 (*P* < 0.01).

## 4. Discussion

This study is the first to evaluate the influence of the enhancement of beef muscles for consumption as fajita meat with phosphate or an alternative functional ingredient. The results from this study show that enhancing various muscles either with phosphate or sodium bicarbonate improved the tenderness, juiciness, flavor liking, and ultimately overall liking compared with non-enhanced muscles. The clean enhancement solution garnered greater tenderness and juiciness scores compared with the phosphate enhancement, regardless of the muscle, but flavor liking did not differ between the two enhancement solutions, resulting in similar overall liking. Despite similar percent pickup, the clean enhancement samples had greater post-processing pH compared with the samples enhanced with phosphate. A greater pH for the clean samples was expected, as sodium bicarbonate possesses a greater buffering capacity in water than sodium phosphate salts [21]. This increase in pH led to lower cooking loss and greater cooked moisture percentage. Ultimately, this resulted in greater consumer juiciness without affecting flavor liking. We believe this likely contributed to the greater consumer tenderness scores of the clean vs. phosphate enhanced samples as well, given the high positive correlation coefficient (r = 0.89) between consumer tenderness and juiciness.

Consumers also detected palatability differences between the muscles in the current study. Huerta-Montauti et al. [9] evaluated several muscles from the beef carcass to find muscles suitable as beef fajita options. Common muscles between the two studies included the outside skirt, inside skirt, and bottom sirloin flap. In the current study, the bottom sirloin flap received greater palatability scores across all traits and was liked the most compared with all the other muscles. Huerta-Montauti et al. [9] found that the bottom sirloin flap and outside skirt were more tender than the inside skirt, but tenderness liking, flavor liking, and overall liking did not differ between these three muscles when papain was either applied alone or in conjunction with blade tenderization [9]. In the current study, the inside round cap was the least tender and the least juicy compared with all the other muscles, which resulted in low overall liking, suggesting this muscle may not be a suitable option for fajita production, even with enhancement. 

Jeremiah et al. [22] assessed the palatability of several major beef muscles, including four common muscles to the current study: the outside skirt, inside round cap, bottom sirloin flap, and flank steak. All the muscles, except the outside skirt were considered slightly tough initially according to trained panelists, while the outside skirt was neither tough nor tender. Of all 33 muscles evaluated by [22], the outside skirt had the most intense flavor but consequently had the second least desirable flavor. The other common muscles had slightly intense flavor with slightly desirable flavor. Of the common muscles between the two studies, the inside round cap had the greatest overall palatability, followed by the flank steak, outside skirt, and bottom sirloin flap. Granted, Jeremiah et al. [22] roasted samples and used trained panelists compared with the untrained panelists used in the current study. In addition, no enhancement was used by [22]. However, their results are essentially reversed from the current findings. Belew et al. [23] determined Warner-Bratzler shear force values of 40 beef muscles, including all five used in the current study. According to their results, the outside skirt was the most tender of all the muscles evaluated, which they attributed to the high fat content of the diaphragm muscle. The bottom sirloin flap was the second most tender of the remaining common muscles, which was scored considerably more tender in the present findings. However, the second most tender according to [23] was the inside round cap, which contradicts the current findings, followed by the flank steak and inside skirt. Belew et al. [23] cooked samples as steaks, which aligns more closely to the cooking methods in the current study, but no enhancement was used.

Both types of enhancement improved all palatability traits regardless of the muscle. Previous findings have shown that the incorporation of various non-meat ingredients increased tenderness, juiciness, and flavor [2,3,5,6,24]. In alignment with the current findings, enhanced beef is typically juicier than non-enhanced beef [2,6]. Enhancement can increase muscle pH and decrease free water, increasing moisture retention [5,24]. Morrow et al. [10] found that enhanced flank steaks had a greater cooked moisture percentage than non-enhanced samples, and consumers scored those enhanced samples as juicier. Flavor liking and overall liking increased in the current study due to enhancement, again aligning with previous results [5,6,24]. According to previous results, sodium chloride can increase saltiness and enhance beef flavor intensity [25]; however, others have speculated that enhancement ingredients, particularly salt, could mask other flavors, including beef intensity [26,27]. Nonetheless, saltiness did not appear to be overwhelming or detrimental in the current results, as flavor liking improved with enhancement. Likewise, Morrow et al. [10] had consumers score flank steaks for saltiness using “Just about right” scales, where 50 was “Just about right”. Their brine solution had a similar salt concentration as the current study. Scores above 50 indicated that the samples were too salty, and scores below 50 suggested the samples were not salty enough. Saltiness was similar between various methods of enhancement (tumbling, injection, or combination), closest to “Just about right” and greater than the non-enhanced flank steak samples, but not to the extent that the enhanced samples were too salty.

## 5. Conclusions

Muscle and enhancement independently influenced tenderness, juiciness, flavor liking, and overall liking. The MSA grade only impacted tenderness but had no effect on other palatability traits. The bottom sirloin flap was liked most overall compared with all the other muscles, while the inside round cap was liked less but did not differ from the inside skirt or flank steak. The samples enhanced with the “clean label” ingredient, sodium bicarbonate, were the most tender and juicy, the samples enhanced with phosphate were intermediate, and the non-enhanced control samples were the least tender and juicy, regardless of muscle or MSA grade. Despite differences in tenderness and juiciness between the enhanced samples, flavor and overall liking were similar between the clean and phosphate-enhanced samples, and both were liked more than the control samples. Enhancement was necessary for acceptable eating quality of the muscles evaluated in this study; however, the inside round cap may not be a suitable fajita option due to significantly reduced tenderness and juiciness compared with the other muscles. Flavor and overall liking were not different between the two enhancement formulations, but tenderness and juiciness varied. These results indicate that a “clean label” enhanced product is possible without compromising cooking yield or consumer satisfaction; in fact, the reverse was true. This may be an important finding for markets in which consumers are sensitive to ingredient labeling and desire a “natural” product.

## Figures and Tables

**Table 1:** Mean, standard deviation, and range of carcass traits (*n* = 22)**.**

Trait	Mean	Standard Deviation	Minimum	Maximum
Dentition	2.5	1.3	0	4
Carcass weight, kg	298.6	47.7	216	388.5
Hump height, mm	114.3	24.2	65	160
Eye muscle area, cm^2^	73.3	11.6	49	90
Rib fat, mm	8.9	3.3	3	18
Ossification	158.6	13.2	140	180
Marbling	456.4	186.4	200	780
Meat color	2.7	0.7	1C	5
Fat color	1.1	1	0	3
pH	5.56	0.07	5.43	5.7
Temperature, °C	5	0.7	3	6.1

**Table 2:** The main effects of muscle and enhancement on the processing characteristics of the Australian beef muscle samples (*n* = 216) ^1^_._

Treatment	Post- Processing pH	Green Weight, kg	Tumbled Weight, kg	Percent Pickup, %
**Muscle**				
Outside skirt	5.78 ^d^	0.29 ^c^	0.33 ^c^	15.9 ^a^
Inside skirt	5.92 ^bc^	0.47 ^b^	0.53 ^b^	12.8 ^b^
Bottom sirloin flap	6.06 ^a^	0.62 ^a^	0.70 ^a^	12.9 ^b^
Flank steak	5.95 ^ab^	0.33 ^c^	0.35 ^c^	7.2 ^d^
Inside round cap	5.81 ^cd^	0.51 ^b^	0.56 ^b^	9.6 ^c^
SEM ^2^	0.429	0.02	0.22	0.49
*P*-value	<0.01	<0.01	<0.01	<0.01
**Enhancement**				
Clean	6.43 ^a^	0.45	0.5	11.8
Phosphate	5.84 ^b^	0.44	0.5	11.5
Control	5.43 ^c^	-	-	-
SEM	0.328	0.012	0.013	0.3
*P*-value	<0.01	0.96	0.96	0.52
*P*-value (muscle × enhancement)	0.16	0.65	0.73	0.1

^a–d^ Within a column, least squares means without a common superscript differ (*P* < 0.05) due to muscle. ^1^ Muscle: outside skirt (*n* = 42), inside skirt (*n* = 42), bottom sirloin flap (*n* = 44), flank (*n* = 44), inside round cap (*n* = 44); Enhancement: *n* = 72/treatment. ^2^ Pooled (largest) SE of least squares means.

**Table 3:** The main effects of muscle and enhancement on the cooking properties (cooking loss and cooking time) of the Australian beef muscle samples (*n* = 216) ^1^**_._**

Treatment	Raw Weight, g	Cooked Weight, g	Cooking Loss, %	Cooking Time, s
**Muscle**				
Outside skirt	279.8 ^d^	196.8 ^c^	29.2 ^a^	379 ^d^
Inside skirt	449.2 ^c^	337.5 ^b^	25.0 ^b^	407 ^d^
Bottom sirloin flap	634.5 ^a^	462.5 ^a^	26.7 ^ab^	822 ^b^
Flank steak	310.6 ^d^	227.1^c^	27.0 ^ab^	556 ^c^
Inside round cap	507.6 ^b^	363.4 ^b^	28.5^a^	986 ^a^
SEM^2^	17.84	12.92	0.99	39
*P*-value	<0.01	<0.01	0.02	<0.01
**Enhancement**				
Clean	459.0 ^a^	341.5 ^a^	25.4 ^b^	621
Phosphate	449.9 ^a^	318.6 ^ab^	29.3 ^a^	620
Control	400.1 ^b^	292.3 ^b^	27.1 ^b^	649
SEM^2^	13.63	9.86	0.76	30
*P*-value	<0.01	<0.01	<0.01	0.71
*P*-value (muscle × enhancement)	0.58	0.36	0.32	0.89

^a–d^ Within a column, least squares means without a common superscript differ (*P* < 0.05) due to muscle. ^1^ Muscle: outside skirt (*n* = 42), inside skirt (*n* = 42), bottom sirloin flap (*n* = 44), flank (*n* = 44), inside round cap (*n* = 44); Enhancement: *n* = 72/treatment. ^2^ Pooled (largest) SE of least squares means.

**Table 4:** The interactive effects (*P* < 0.01) of muscle and enhancement on the cooked moisture of the Australian beef muscle samples (*n* = 216) ^1^**_._**

Muscle	Clean	Phosphate	Control
Outside skirt	62.5 ^cdef^	57.8 ^g^	50.6 ^h^
Inside skirt	66.4 ^a^	62.1 ^def^	55.5 ^g^
Bottom sirloin flap	64.8 ^abc^	62.8 ^cde^	57.1 ^g^
Flank steak	64.2 ^abcd^	61.8 ^ef^	57.7 ^g^
Inside round cap	65.3 ^ab^	64.0 ^bcde^	60.3 ^f^
SEM ^2^	0.97		

^a–h^ Within all columns and rows, least squares means without a common superscript differ (*P* < 0.05). ^1^ Muscle: outside skirt (*n* = 42), inside skirt (*n* = 42), bottom sirloin flap (*n* = 44), flank (*n* = 44), inside round cap (*n* = 44); Enhancement: *n* = 72/treatment. ^2^ Pooled (largest) SE of least squares means.

**Table 5:** The demographic characteristics of consumers (*n* = 360) who participated in the consumer sensory panels at Texas Tech University in Lubbock, TX, USA.

Characteristic	Response	% of Consumers
Age group	<20	5.8
	20–29	22.5
	30–39	31.9
	40–49	14.2
	50–59	13.9
	>60	11.7
Gender	Male	47.0
	Female	53.1
Ethnic origin	African American	1.7
	Asian	0.0
	Caucasian/white	49.0
	Hispanic	48.2
	Native American	0.6
	Other	0.6
Household size (adults)	1	9.5
	2	64.6
	3	18.1
	4	4.5
	5	2.8
	6+	0.6
Household size (children)	0	47.0
	1	11.1
	2	22.5
	3	13.9
	4	3.1
	5	2.5
Annual household income	<$20,000	9.8
	$20,000–$50,000	24.9
	$50,001–$75,000	23.0
	$75,001–$100,000	19.3
	>$100,000	
Level of education	Non-high school graduate	5.3
	High school graduate	22.9
	Some college/technical school	32.4
	College graduate	26.8
	Post-college graduate	12.6
Beef consumption	Daily	8.9
	4–5 times a week	33.2
	2–3 times a week	40.7
	Weekly	11.7
	Every other week	4.7
	Monthly	0.8
Preferred beef degree of doneness	Rare	4.8
	Medium-rare	35.4
	Medium	25.6
	Medium-well	23.0
	Well-done	11.2

**Table 6:** The main effects of muscle, enhancement, and Meat Standards Australia (MSA) grade on consumer scores (*n* = 360) for tenderness, juiciness, flavor liking, overall liking, composite score (MQ4), and satisfaction of the Australian beef muscles prepared for fajitas.

Treatment	Tenderness ^1^	Juiciness ^1^	Flavor Liking ^1^	Overall Liking^1^	MQ4 ^2^	Satisfaction ^3^
**Muscle ^4^**						
Outside skirt	64.0 ^b^	60.0 ^b^	54.7 ^b^	56.5 ^b^	58.6 ^b^	3.22 ^b^
Inside skirt	59.0 ^c^	54.4 ^c^	52.5 ^b^	53.7 ^bc^	55.1 ^b^	3.13 ^bc^
Bottom sirloin flap	73.7 ^a^	66.7 ^a^	67.5 ^a^	68.6 ^a^	69.6 ^a^	3.65 ^a^
Flank steak	57.3 ^c^	51.2 ^c^	54.8 ^b^	55.0 ^bc^	55.3 ^b^	3.16 ^bc^
Inside round cap	49.2 ^d^	44.7 ^d^	52.2 ^b^	51.2 ^c^	50.3 ^c^	3.01 ^c^
SEM ^7^	1.8	1.8	1.7	1.7	1.5	0.058
*P*-value	<0.01	<0.01	<0.01	<0.01	<0.01	<0.01
**Enhancement ^5^**						
Clean	70.8 ^a^	64.1 ^a^	61.9 ^a^	64.0 ^a^	65.5 ^a^	3.48 ^a^
Phosphate	64.5 ^b^	59.4 ^b^	62.9 ^a^	63.2 ^a^	63.1 ^a^	3.43 ^a^
Control	46.7 ^c^	42.7 ^c^	44.2 ^b^	43.9 ^b^	44.7 ^b^	2.78 ^b^
SEM ^7^	1.4	1.5	1.3	1.3	1.3	0.46
*P*-value	<0.01	< 0.01	<0.01	<0.01	<0.01	<0.01
**MSA Grade ^6^**						
5*—Premium	58.7 ^b^	55.3	54.8	55.1	56.1	3.20
4*—Classic	64.3 ^a^	58.1	57.8	58.8	60.1	3.21
3*—Selected	60.5 ^ab^	54.7	56.1	56.6	57.5	3.24
2—Unsatisfactory	59.1 ^b^	53.5	56.7	57.5	57.4	3.28
SEM^7^	2.0	2.1	1.9	1.9	1.8	0.065
*P*-value	0.04	0.29	0.46	0.29	0.17	0.67

^a–d^ Within a column, least squares means without a common superscript differ (*P* < 0.05) due to muscle. ^1^ Scores: 0 mm = not tender, not juicy, dislike flavor extremely, dislike overall extremely; 100 mm = very tender, very juicy, like flavor extremely, like overall extremely. ^2^ MQ4 = tenderness*0.3 + juiciness*0.1 + flavor liking* 0.3 + overall liking*0.3. ^3^ Satisfaction score: 2 = unsatisfactory, 3 = good everyday quality, 4 = better than everyday quality, and 5 = premium quality. ^4^ Muscle: outside skirt (*n* = 42), inside skirt (*n* = 42), bottom sirloin flap (*n* = 44), flank (*n* = 44), and inside round cap (*n* = 44). ^5^ Enhancement: *n* = 72/treatment. ^6^ MSA grade: premium (*n* = 68), classic (*n* = 50), selected (*n* = 68), and unsatisfactory (*n* = 30). ^7^ Pooled (largest) SE of least squares means.

**Table 7:** Pearson correlation coefficients of relationships between composition, cooking traits, and consumer eating quality scores.

Attribute	Post- Processing pH	Percent Pickup	Moisture	Cooking Time	Cooking Loss
Tenderness	0.53 **	0.65 **	0.34 **	0.00	−0.16 *
Juiciness	0.47 **	0.62 **	0.26 **	−0.04	−0.14 *
Flavor liking	0.43 **	0.58 **	0.32 **	0.24 **	0.04
Overall liking	0.46 **	0.60 **	0.33 **	0.16 *	−0.03

** Correlation coefficient differs from 0 (*P* < 0.01). * Correlation coefficient differs from 0 (*P* < 0.05).

**Table 8:** Pearson correlation coefficients of relationships between consumer eating quality scores.

Attribute	Tenderness	Juiciness	Flavor Liking	Overall Liking	MQ4
Juiciness	0.89 **				
Flavor liking	0.80 **	0.80 **			
Overall liking	0.88 **	0.87 **	0.96 **		
MQ4	0.94 **	0.91 **	0.95 **	0.98 **	
Satisfaction	0.83 **	0.82 **	0.91 **	0.93 **	0.92 **

** Correlation coefficient differs from 0 (*P* < 0.01).
